# Associations between Perceived Neighborhood Walkability and Walking Time, Wellbeing, and Loneliness in Community-Dwelling Older Chinese People in Hong Kong

**DOI:** 10.3390/ijerph14101199

**Published:** 2017-10-09

**Authors:** Ruby Yu, Osbert Cheung, Kevin Lau, Jean Woo

**Affiliations:** 1 Department of Medicine and Therapeutics, Faculty of Medicine, the Chinese University of Hong Kong, Hong Kong, China; osbertcheung@cuhk.edu.hk (O.C.); jeanwoowong@cuhk.edu.hk (J.W.); 2 CUHK Jockey Club Institute of Ageing, the Chinese University of Hong Kong, Hong Kong, China; kevinlau@cuhk.edu.hk; 3 Institute of Future Cities, the Chinese University of Hong Kong, Hong Kong, China; 4 Institute of Environment, Energy and Sustainability, the Chinese University of Hong Kong, Hong Kong, China

**Keywords:** perceived neighborhood walkability, walking time, physical activity, wellbeing, life satisfaction, happiness, sense of purpose and meaning in life, loneliness

## Abstract

This study examined the cross-sectional associations between perceived neighborhood walkability and walking time, physical activity, wellbeing, and loneliness, and examined which components of walkability were most strongly associated with better wellbeing and less loneliness in older adults. Participants were community-dwelling Chinese adults aged 60+ (*n* = 181). Walkability was measured using nine items selected from the Chinese version of the abbreviated Neighborhood Environment Walkability Scales (NEWS) and NEWS for Chinese Seniors. Outcomes were walking time, physical activity, wellbeing (life satisfaction, happiness, sense of purpose and meaning in life), and loneliness. The mean age of the participants was 71.7 ± 7.8 years. Walkability was positively associated with walking time (*p* = 0.001, *p* for trend <0.001) but not with physical activity. After adjusting for socio-demographic characteristics, health conditions, lifestyle, and negative life events, those who perceived their neighborhoods as walkable had higher scores for life satisfaction (*p* = 0.002) and happiness (*p* = 0.002), and lower scores for loneliness (*p* = 0.019), compared with those who perceived their neighborhoods as less walkable. However, perceived neighborhood walkability was not associated with sense of purpose and meaning in life. Among components of walkability, land use mix-access, infrastructure and safety for walking, and traffic safety showed the strongest associations with the measures of wellbeing. The results of this study support the importance of neighborhood walkability for health behavior and wellbeing of older adults. The wellbeing of older adults may be enhanced through the improvement of land use mix-access, infrastructure for walking, and traffic safety.

## 1. Introduction

As worldwide life expectancy increases, the issue of maintaining wellbeing at an advanced age is growing in importance, as evidence suggests that active engagement with life and positive wellbeing are protective factors for health as people age [1,2,3,4]. Studies of older people show that subjective wellbeing, in terms of life satisfaction, is affected by the individuals’ state of physical health [5], socioeconomic status, as well as social and family relationships [6]; nevertheless, neighborhood environments may also affect the wellbeing of older adults.

Neighborhood environments are being increasingly recognized as factors that influence health and wellbeing. Neighborhoods designed to be more walkable can encourage residents to walk more and may promote physical activity and social connectedness, which in turn has beneficial effects on health and wellbeing, particularly in older people. This is because older people may be more susceptible to neighborhood effects due to a combination of increasing frailty, functional decline [7] and diminishing social networks [8], which could lead to a greater dependence on locally provided services and amenities. In a cross-sectional study carried out in Belgium, older adults who lived in the most walkable neighborhood (defined by residential density, street connectivity, and land use mix-diversity) walked more frequently every week than those in the least walkable neighborhood [9]. Notably, a recent multi-country study based on data from 14 cities worldwide reported that more walkable neighborhoods (defined by residential density, intersection density, public transport density, and number of parks) were associated with a higher level of physical activity [10]. There is some evidence to support the association between neighborhood walkability and health outcomes. For example, a recent time-series analysis carried out in Canada found that more walkable neighborhoods were associated with decreased prevalence of overweight and obesity and decreased incidence of diabetes over 12 years [11]. A previous study in Washington noted that neighborhood walkability was inversely associated with depression in older men [12].

Although research supports the importance of neighborhood walkability for health and wellbeing, this line of research is still in its infancy, and the role of walkability on subjective wellbeing has been relatively unstudied. Previous studies have mostly been conducted in Western countries; it is still not known whether walkability has any role in the wellbeing of Hong Kong Chinese, who live in much denser environments in terms of population and destinations, and who may differ significantly from Caucasians in terms of culture and lifestyle. In this study, we examined the associations of walkability with walking time, physical activity, subjective wellbeing, and loneliness. We also aimed to examine which components of walkability were most strongly associated with better wellbeing and less loneliness in older adults.

## 2. Materials and Methods

### 2.1. Design and Participants

To examine the relationship between neighborhood characteristics, health, wellbeing, and loneliness, a survey was conducted in two selected districts of Hong Kong, including Sha Tin and Tai Po. The two districts are located in the New Territories of Hong Kong, with their population estimated at 659,794 and 303,926 in 2016, respectively [13]. They were chosen for the study because they have a mix of neighborhood types ranging from mixed-use town centers to areas covering traditional villages. On the whole, seven neighborhoods were chosen, namely Sha Tin Town Centre, Lek Yuen & Wo Che, Ma On Shan Town Centre, Yee Fu & Kwong Fuk, Tai Po Centre, Tai Po Hui & Old Market, and Lam Tsuen Valley, which are represented by a range of typical housing types in different settings (private/subsided/public housing in town centers, tenement housing in old urban core, village house in low-to mid-density areas). For example, Sha Tin Town Centre, Ma On Shan Town Centre, and Tai Po Centre are areas which accommodate private, subsided, and public housing supplemented with commercial and open space to form a mixed-use development pattern. Lek Yuen & Wo Che and Yee Fu & Kwong Fuk are areas which accommodate predominantly public rental housing supported by essential infrastructure and community facilities. Tai Po Hui & Old Market are clustered around tenement housing. Lam Tsuen Valley is situated in the west of Tai Po, an area covering both traditional villages and new residential housing. For this study, a neighborhood was defined as a spatial unit within which urban residents share similar socioeconomic and cultural identities. Neighborhood boundaries were delineated using the government web map portal, GeoInfo Map (http://www1.map.gov.hk/gih3/view/index.jsp). Major roads and waterways served as barriers to movement and communication and therefore served as logical boundaries of the neighborhood.

Three hundred and one community-dwelling Chinese adults aged 60 years and older were recruited in the survey between June and August 2017. Participants were recruited by placing recruitment notices in housing estates and elderly community centres. Several talks were also given at the centres explaining the purpose and interviews to be carried out. An age-stratified sample of volunteers was recruited, so that approximately 50% of the participants would be aged 60–69, 30% would be aged 70–79, and 20% would be aged 80 years and older, according to the age structure of the mid-year population (aged 60 years and older) of Hong Kong, 2016. To be eligible, participants needed to be aged 60 years and older, able to walk, and able to speak Cantonese. A team of trained research assistants administered the questionnaire for each participant face-to-face. The initial phase of the study did not collect data on walkability. Therefore, we excluded 120 participants whose walkability data was not available, yielding a study of 181 participants (living in five of the selected neighborhoods: Sha Tin Town Centre, Yee Fu & Kwong Fuk, Tai Po Centre, Tai Po Hui & Old Market, and Lam Tsuen Valley) for analysis in the present study. All participants gave written consent, and the study was approved by the Survey and Behavioral Research Ethics Committee of the Chinese University of Hong Kong (026-16). The study was performed in compliance with the declaration of Helsinki.

### 2.2. Measures

#### 2.2.1. Perceived Neighborhood Walkability

Perceived neighborhood walkability was measured using items selected from the Chinese version of the abbreviated Neighborhood Environment Walkability Scale (Chinese NEWS-A) [14] and NEWS for Chinese Seniors (NEWS-CS) [15]. The Chinese NEWS-A consists of 54 separate items distributed among eight sub-scales: (a) residential density with six items; (b) proximity to nonresidential land uses (i.e., land uses mix-diversity) with 23 items; (c) ease of access to nonresidential uses (i.e., land use mix-access) with six items; (d) street connectivity with three items; (e) infrastructure and safety for walking with six items; (f) aesthetics with four items; (g) pedestrian traffic safety with three items; and (h) safety from crime with three items. Residential density items are rated on a 5-point Likert scale. Land use mix-diversity is rated by the walking distance from home to various types of stores and facilities, with responses ranging from a walk from one to five minutes to a walk of >30 min. Items on the other sub-scales are rated on a 4-point Likert scale from 1 (strongly disagree) to 4 (strongly agree). Higher scores indicate more walkable. NEWS-CS was developed based on the original English and Chinese translation of NEWS-A. NEWS-CS consists of 76 items, which includes 52 out of 54 items from NEWS-A, in their original or slightly modified form, and 24 additional items describing the environment features relevant to senior residents. The reliability and validity of the Chinese NEWS-A (subscale reliability intraclass correlation coefficients (ICCs): 0.57–0.99; average validity ICC: 0.46) [14] and NEWS-CS (subscale reliability ICCs: 0.37–0.77; standardized root mean squared residual: 0.067; root mean square error of approximation: 0.037) have been demonstrated [15].

##### Selection of Items for This Study

A panel of experts from the fields of geriatric medicine, public health, and urban planning reviewed Chinese NEWS-A and NEWS-CS, and developed a reduced version to measure perceived neighborhood walkability. The reduced version has a total of nine items: two items from land use mix-access, one item from street connectivity, three items from infrastructure and safety for walking, one item from aesthetics, one item from pedestrian traffic safety, and one item from safety from crime. The reduced version was scored using the original 4-point scoring system from 1 (strongly disagree) to 4 (strongly agree). The total walkability score was computed by taking the average of the nine items, where items 2, 8, and 9 were reverse scored. Possible scores range from 1 to 4, with 1 meaning less walkable and 4 meaning more walkable.

##### Reliability of the Reduced Version

The nine-item reduced version has been validated against the corresponding sub-scales of the Chinese NEWS-A in a sample of 46 community-dwelling older adults aged 60–91 years, of whom 54.3% were women and 43.6% had some secondary or tertiary education. Cronbach’s alphas of the reduced version (0.776) and the Chinese NEWS-A (0.652) were similar. In addition, correlations between individual items of the reduced version and the corresponding items of the Chinese NEWS-A were moderate to moderately-strong (*r* = 0.420–0.723). With the exception of the aesthetics sub-scale, there is no difference between the mean score of each item of the two versions (*p* = 0.284–0.609) (Table A1).

#### 2.2.2. Walking Time

Walking in the past seven days was measured with two separate items extracted from the Physical Activity Scale for the Elderly (PASE) [16,17]. Participants were asked about how frequently they walked outside their home for any purpose (0 day, 1–2 days, 3–4 days, and 5–7 days) and the duration of these walks (<1 h/day, 1–2 h/day, 3–4 h/day, >4 h/day). Time spent on walking in the past seven days (hours) was calculated by multiplying the median values of the frequency and duration categories. Examples for purposes of the walk were given, including for fun or exercise, walking to work, walk the dog, groceries shopping, throw out the garbage, go to have dim sum, go shopping etc.

#### 2.2.3. Physical Activity (Excluding Walking)

Physical activity was measured using two separate items. Participants were asked how frequently they carried out physical activity (0 day/week, 1 day/week, 2 days/week, 3 days/week, 4 days/week, 5 days/week, 6 days/week and 7 days/week) and the duration of these sessions (<20 min/day, 20–59 min/day, 1–2 h/day, 2–3 h/day, and >3 h/day). Time spent on physical activity (hours/week) was calculated by multiplying the frequency and the median values of the duration categories. Physical activity was defined as any physical activities for sport, exercise or recreation, including hiking, but not walking nor shopping. Examples for physical activities were given, including Tai Chi, jogging, hiking, swimming and using the fixed fitness equipment in fitness stations located in parks designed specifically for seniors.

#### 2.2.4. Subjective Wellbeing and Loneliness

Subjective wellbeing was quantified in terms of life satisfaction, happiness, and sense of purpose and meaning in life. Life satisfaction was measured with a single item, “Overall, how satisfied are you with life as a whole these days?” which participants rated on a scale from 0 as “not at all satisfied” to 10 as “completely satisfied” [18]. Happiness was measured with a single item, “How happy did you feel yesterday?” which participants rated on a scale from 0 as “not at all happy” to 10 as “completely happy” [18]. Sense of purpose and meaning in life was measured with a single item, “Do you feel your life has an important purpose or meaning?” which participants rated on a scale from 0 as “not at all worthwhile” to 10 as “completely worthwhile” [18]. Loneliness was assessed using the six-item De Jong Gierveld Loneliness Scale [19]. The scale consists of three items assessing emotional loneliness: “I experience a general sense of emptiness,” “I miss having people around,” and “I often feel rejected,” and three items assessing social loneliness: “There are plenty of people I can rely on when I have problems,” “There are many people I can trust completely,” and “There are enough people I feel close to.” The response categories were “yes,” “more or less,” and “no,” collapsed to form a binary (0/1) attribute (i.e., for emotional loneliness, “yes” and “more or less” = 1, “no” = 0; for social loneliness, “no” and “more or less” = 1, “yes” = 0). The overall loneliness score was computed by taking the sum of the six items. Possible scores range from 0 to 6, where 0 means “no loneliness” and 6 indicates “severe loneliness.” The Chinese version of the scale has been validated and shown to be reliable [19,20].

#### 2.2.5. Covariates

Socio-demographic characteristics (age, sex, marital status, education, employment, income, type of housing, length of residence, and living arrangement), health conditions (number of doctor-diagnosed chronic diseases and number of prescribed medications), and lifestyle (smoking and alcohol intake) were collected. Participants were also asked to report the occurrence of any of the seven negative life events extracted from the Holmes and Rahe Stress Scale in the past year. These include the serious illness or accident of spouse/partner, death of spouse/partner/other close relative/close friend, separation from child/close friend/other relative upon whom participant depends, given up important hobby or activity, serious financial trouble and/or move or change in residence [21], and the loss of pet, as adopted from the Osteoporotic Fractures in Men (MrOS) study [22]. Each type of negative life event was counted only once per participant.

### 2.3. Data Analysis

We grouped the participants based on tertiles of the walkability score: lowest (<3.1), middle (3.1–3.6), and highest (>3.6). Participants’ characteristics, walking time, and physical activity levels were compared across tertiles of the walkability score using analysis of variance (ANOVA) for continuous variables and chi-square tests for categorical variables. Tests for trends were also performed as appropriate. Multivariable linear regression models were used to investigate the associations of perceived neighborhood walkability with life satisfaction, happiness, sense of purpose and meaning in life, and loneliness, which were treated as continuous variables. To examine which components of walkability were most strongly associated with measures of subjective wellbeing and loneliness, the response “strongly disagree,” “disagree,” “agree,” and “strongly agree” were coded into three dummy variables with “strongly agree” as the reference category (for items 1, 3–7) and “strongly disagree” as the reference category (for items 2, 8, 9) and the regression models were repeated. Each model was analyzed with adjustment for individual characteristics (age, sex, marital status, education, employment, income, type of housing, length of residence, number of doctor-diagnosed chronic diseases, number of prescribed medications, smoking, alcohol intake, and negative life events). All statistical tests were two sided. A *p* value of less than 0.05 was considered statistically significant. Estimates based on fewer than 30 observations are considered unreliable. All statistical analyses were performed using the statistical package SPSS, version 24 (SPSS Inc., Chicago, IL, USA).

## 3. Results

The descriptive characteristics of the study population according to walkability tertiles are reported in Table 1. The mean age of the 181 participants was 71.7 ± 7.8 years (range 60–95 years), 48.1% were women, the majority were married (71.0%), and 50.3% had some secondary or tertiary education. The mean length of residence was 23.7 ± 13.6 years (range 0.5–78 years). The mean walkability score was 3.3 ± 0.5 (range 1.7–4.0) and the lowest and highest tertile values were 3.1 and 3.6, respectively. Participants in the highest tertile had higher income (*p* = 0.007, *p* for trend = 0.003), lived more often in private high-rise housing (*p* < 0.001), and had longer length of residence (*p* = 0.006) than those in the middle and lowest tertiles.

Table 2 presents the score and percentage of response of each individual walkability item. The highest mean item score was recorded for item 4, followed by items 7 and 3. The lowest scores were recorded for items 5, 6, and 8. No items were missing.

Table 3 presents the walking time and physical activity levels according to tertiles of perceived neighborhood walkability. On average, 76.8% of participants reported that they walked in their neighborhoods for at least 5.25 h in the past seven days. Participants who perceived their neighborhoods to be more walkable (the highest tertile) reported more weekly hours spent on walking than participants who perceived their neighborhoods to be less walkable (the lowest tertile) (*p* = 0.001, *p* for trend < 0.001). The pattern remained significant after adjusting for age, sex, marital status, education, employment, income, type of housing, length of residence, number of doctor-diagnosed chronic diseases, number of prescribed medications, smoking, alcohol intake, and negative life events. However, walkability was not associated with physical activity.

Table 4 presents the associations of perceived neighborhood walkability with measures of subjective wellbeing and loneliness. The scores for life satisfaction, happiness, sense of purpose and meaning in life, and loneliness were 7.7 ± 2.0, 7.9 ± 2.0, 8.1 ± 1.7 and 1.6 ± 1.6, respectively. Participants who perceived their neighborhoods to be more walkable (the highest tertile) reported higher levels of life satisfaction (*p* = 0.002) and happiness (*p* = 0.002), and lower levels of loneliness (*p* = 0.019), after adjusting for the same list of covariates. However, walkability was not associated with sense of purpose and meaning in life.

Table 5 presents the associations of each component of walkability with measures of wellbeing and loneliness. Land use mix-access was positively associated with life satisfaction and happiness (all *p* < 0.05), but not with sense of purpose and meaning in life. Street connectivity was positively associated with happiness (*p* < 0.05) but not with life satisfaction or sense of purpose and meaning in life. Infrastructure and safety for walking was positively associated with happiness (all *p* < 0.05), but not with life satisfaction or sense of purpose and meaning in life. Aesthetics was positively associated with happiness (*p* = 0.025). Traffic safety was associated with each measure of wellbeing (all *p* < 0.05). Safety from crime was positively associated with happiness (*p* = 0.046) but not with life satisfaction or sense of purpose and meaning in life. No associations were observed between any of the walkability components and loneliness after multivariable adjustments.

## 4. Discussion

This study examined the associations between perceived neighborhood walkability and walking time, physical activity, subjective wellbeing, and loneliness in older Chinese adults. The results showed that perceived neighborhood walkability was positively associated with walking time, but not with physical activity. Perceived neighborhood walkability was significantly associated with better life satisfaction, happiness, and less loneliness, with land use mix-access, infrastructure for walking, and traffic safety showing the strongest associations.

To date, several studies have investigated the relationship between perceptions of neighborhood environment characteristics and walking behaviors, as walking is the dominant physical activity carried out by older adults during their leisure time. These studies, however, were predominantly conducted in Western countries, where the built environment can differ substantially from Asian regions, particularly Hong Kong, which is one of the most densely populated cities in the world. Using a subjectively measured walkability, we found that participants who lived in the most walkable neighborhood reported more weekly hours of walking than those who lived in the least walkable neighborhood. This finding adds further evidence to the findings of previous studies showing that neighborhood walkability, either objectively (as measured by a walkability index) or subjectively measured, is positively associated with walking time [23,24].

Although walkability was associated with walking time, our findings did not find any association between perceived walkability and physical activity (excluding walking). This finding is consistent with previous studies done on Australian and Belgian older adults, where walkability was not associated with any measures of recreational physical activity [9,25]. Findings from a systematic review also point to associations between walkability and walking for transport, but not to other types of physical activity [26]. This was to be expected because walkability measures (e.g., NEWS score, walkability index) were designed to measure walking for transport or other purposes but not specifically for physical activity. It is also possible that physical activity patterns in older adults are related to psychosocial factors (e.g., psychosocial support from relatives and friends) in addition to environmental factors. A previous study in Washington demonstrated that a combination of environmental and psychosocial factors was the best in explaining physical activity among older adults [27]. Nevertheless, in a recent multi-country study, walkability defined by residential density, intersection density, public transport density, and number of parks was associated with total moderate to vigorous intensity physical activity in adults aged 18–66 years [10]. The discrepancy may be partially explained by the difference in characteristics between the two studies. This discrepancy also raises the question of whether the influence of other specific built environmental attributes differs by age group. Therefore, further studies are needed to explore the specific environmental attributes characterizing the ability of older adults to participate in physical activities in a neighborhood.

Our findings also indicated that walkability was associated with better life satisfaction, happiness, and less loneliness. This finding is consistent with previous studies linking walkability with perceived quality of life [28] and depression [12]; and studies associating built environment attributes (e.g., housing quality, perceived neighborhood greenness) with psychological wellbeing [29,30]. Although the pathway linking neighborhood walkability to wellbeing is unclear and the cross-sectional associations observed in most of these studies cannot infer causality, increased walkability is expected to increase mobility (e.g., increased walking time), which may help to encourage a sense of environmental mastery and autonomy because it enables individuals to access activities and essential services, and thus improving an individual’s wellbeing. A previous study showed that older people considered environmental mastery and autonomy to be most important for their life satisfaction [31]. In addition, walkable neighborhoods are also expected to promote a sense of local identity and social connection because they enable individuals to interact and to be involved socially, which in turn will enhance wellbeing. Our supplementary analysis revealed that perceived walkability was positively associated with sense of community (data not shown), suggesting that these behaviors are likely to occur more often in areas perceived to be more walkable. In addition, sense of community was associated with life satisfaction and happiness (data not shown). These results suggest a potential mediating role of sense of community in the relationship between walkability and wellbeing. Indeed, a growing body of literature is establishing walkability as an important determinant of social capital [32,33], which is an important factor of wellbeing [34]. A previous study also showed that sense of community mediated the relationship between walkability and wellbeing [28].

This study also examined which components of walkability were most strongly associated with better wellbeing and less loneliness in older adults. We found that all components were associated with at least one measure of wellbeing, with land use mix-access, infrastructure for walking, and traffic safety showing the strongest associations. These results are consistent with the findings in the literature. For example, a Gallup study has also found that convenient public transportation and easy access to cultural and leisure facilities shows the strongest correlation with happiness [35]. However, our findings demonstrated that the associations between street connectivity and safety from crime with measures of wellbeing were modest compared to other components. Perhaps the selected neighborhoods in this study had limited variability in street connectivity (except Lam Tsuen Valley, where the score of street connectivity was significantly lower compared to other neighborhoods) and safety from crime, which may in part explain the findings.

Our finding with regard to the association between walkability and loneliness was also significant, after adjusting for covariates. This is an important finding because loneliness is highly prevalent among older adults, with a significant impact on elderly health and wellbeing [36]. Previous studies have also found that perceived neighborhood walkability and safety are associated with depression in older adults [12,37]. Therefore, the findings of this study together with current available literature lend support to the concept that walkability may be considered an important factor associated with wellbeing in older adults, and underscores the importance of creating walkable neighborhoods as one key strategy in tackling mental problems in older adults.

Our findings are relevant to policymakers, assisting them in the development of walkable neighborhoods. Walkability involves more than just providing sidewalks—the walking environment should also be comfortable and healthy so that the benefits of walking can be maximized [38]. In high-density cities like Hong Kong, street connectivity is generally good in physical sense but the design of the walking environment requires more attention on the human scale [39]. For example, shading in the form of awnings and street trees is important for preventing pedestrians’ exposure to intense summer sun and hence relieving heat stress [40]. A diverse walking environment can also offer opportunities for pedestrians to avoid places with poor air quality and seek enjoyment during their walk [41].

There are some limitations to this study. Because of the cross-sectional nature of the study, no temporal or causal relationships can be assessed. The results were subject to selection bias as sociable people might be more likely to participate in this study. In addition, due to the small sample size, mediation analyses were not performed. Finally, this study used the measure of perceived neighborhood walkability; objective measures of walkability such as block size and street widths are needed regarding how the neighborhood walkability affects wellbeing in older adults. In spite of these limitations, the strengths of this study include the use of a reduced version of the NEWS tested for reliability, the use of different aspects of subjective wellbeing including life satisfaction, happiness, sense of purpose and meaning in life, and loneliness, and the inclusion of socio-demographic characteristics, health status, lifestyle, and negative life events in the analyses.

## 5. Conclusions

The results of this study support the importance of neighborhood walkability for the health behavior and wellbeing of older adults. The wellbeing of older adults may be enhanced through the improvement of land use mix-access, infrastructure for walking, and traffic safety. Future studies on walkability will need to combine perceived neighborhood walkability together with objective measurements of neighborhood environmental quality to better understand the role of neighborhood walkability on health and wellbeing in older adults, and to examine what are the mechanisms through which older adults perceive more satisfaction, happiness, and meaning, and less loneliness in their life.

## Figures and Tables

**Table 1:** Characteristics of the study population, overall and by walkability tertiles (*n* = 181).

Variable	All Participants	Perceived Neighborhood Walkability		
		Lowest Tertile	Middle Tertile	Highest Tertile
	(*n* = 181)	(*n* = 56)	(*n* = 68)	(*n* = 57)
Age, years, mean ± SD	71.72 ± 7.81	71.84 ± 7.51	71.31 ± 7.96	72.09 ± 8.04
Age group, *n* (%)				
60–69	89 (49.17)	26 (46.43)	39 (57.35)	24 (42.11)
70–79	56 (30.94)	22 (39.29)	14 (20.59)	20 (35.09)
≥80	36 (19.89)	8 (14.29)	15 (22.06)	13 (22.81)
Sex, *n* (%)				
Men	94 (51.93)	27 (48.21)	32 (47.06)	35 (61.40)
Women	87 (48.07)	29 (51.79)	36 (52.94)	22 (38.60)
Marital status, *n* (%)				
Never/widowed/divorced/separated	52 (29.05)	31 (56.36)	53 (79.10)	43 (75.44) *
Married	127 (70.95)	24 (43.64)	14 (20.90)	14 (24.56)
Education level, *n* (%)				
Uneducated/pre-school/primary education	90 (49.72)	31 (55.36)	37 (54.41)	22 (38.60)
Secondary/tertiary education	91 (50.28)	25 (44.64)	31 (45.59)	35 (61.40)
Employment, *n* (%)				
Unemployed	163 (90.56)	48 (85.71)	62 (92.54)	53 (92.98)
Employed	17 (9.44)	8 (14.29)	5 (7.46)	4 (7.02)
Income, Hong Kong Dollars, *n* (%)				
<4000	50 (28.09)	22 (40.00)	21 (31.82)	7 (12.28) **^,†^
4000–7999	56 (31.46)	13 (23.64)	24 (36.36)	19 (33.33)
≥8000	72 (40.45)	20 (36.36)	21 (31.82)	31 (54.39)
Housing type, *n* (%)				
Private high-rise housing	61 (33.70)	4 (7.14)	21 (30.88)	36 (63.16) ***
Tenement housing	30 (16.57)	7 (12.50)	18 (26.47)	5 (8.77)
Subsidized housing	30 (16.57)	5 (8.93)	15 (22.06)	10 (17.54)
Public housing	28 (15.47)	9 (16.07)	13 (19.12)	6 (10.53)
Village housing	32 (17.68)	31 (55.36)	1 (1.47)	0 (0.00)
Length of residence, years, mean ± SD	23.66 ± 13.63	23.94 ± 18.37	24.34 ± 11.61	22.57 ± 10.13
Length of residence, years, *n* (%)				
<10	36 (19.89)	17 (30.36)	10 (14.71)	9 (15.79) **
10–19	22 (12.15)	4 (7.14)	10 (14.71)	8 (14.04)
20–29	44 (24.31)	12 (21.43)	14 (20.59)	18 (31.58)
30–39	64 (35.36)	13 (23.21)	30 (44.12)	21 (36.84)
≥40	15 (8.29)	10 (17.86)	4 (5.88)	1 (1.75)
Living arrangement, *n* (%)				
Living alone	31 (17.13)	13 (23.21)	11 (16.18)	7 (12.28)
Living with others	150 (82.87)	43 (76.79)	57 (83.82)	50 (87.72)
No. of doctor-diagnosed chronic diseases, *n* (%)				
0	27 (14.92)	7 (12.50)	9 (13.24)	11 (19.30)
1–4	129 (71.27)	38 (67.86)	53 (77.94)	38 (66.67)
≥5	25 (13.81)	11 (19.64)	6 (8.82)	8 (14.04)
No. of prescribed medications, *n* (%)				
0	37 (20.44)	11 (19.64)	13 (19.12)	13 (22.81)
1–4	107 (59.12)	30 (53.57)	44 (64.71)	33 (57.89)
≥5	37 (20.44)	15 (26.79)	11 (16.18)	11 (19.30)
Current smoker, *n* (%)				
No	168 (92.82)	51 (91.07)	64 (94.12)	53 (92.98)
Yes	13 (7.18)	5 (8.93)	4 (5.88)	4 (7.02)
Alcohol drinker, *n* (%)				
No	151 (83.43)	43 (76.79)	60 (88.24)	48 (84.21)
Yes	30 (16.57)	13 (23.21)	8 (11.76)	9 (15.79)
No. of negative life events, *n* (%)				
0	93 (51.67)	22 (39.29)	37 (54.41)	34 (60.71)
≥1	87 (48.33)	34 (60.71)	31 (45.59)	22 (39.29)
Walkability, mean ± SD	3.25 ± 0.53	/	/	/
walkability (by neighborhood), mean ± SD				
Shatin Town Centre	3.68 ± 0.29	/	/	/
Yee Fu & Kwong Fuk	3.27 ± 0.37	/	/	/
Tai Po Centre	3.58 ± 0.32	/	/	/
Tai Po Hui & Old Market	3.19 ± 0.44	/	/	/
Lam Tsuen Valley	2.48 ± 0.32	/	/	/

* *p*-value < 0.05, ** *p*-value < 0.01, *** *p*-value < 0.001, ^†^ *p*-value for trend < 0.01.

**Table 2:** Descriptive statistics of perceived neighborhood walkability (*n* = 181).

Subscale and Individual Item		Response (%)					
	Missing Response	Strongly Disagree	Disagree	Agree	Strongly Agree	Median	Mean
*Land use mix-access*							
Item 1. There are many places to go within walking distance at my home	0	12.71	9.39	18.23	59.67	4	3.25
Item 2. The streets in my neighborhood are hilly, making my neighborhood difficult to walk in *	0	48.07	29.28	16.02	6.63	3 *	3.19 *
*Street connectivity*							
Item 3. There are many alternative routes for getting from place to place in my neighborhood	0	5.52	3.31	27.07	64.09	4	3.50
*Infrastructure and safety for walking*							
Item 4. There are sidewalks on most of the streets in my neighborhood	0	1.10	2.21	24.86	71.82	4	3.67
Item 5. There are covered bridges in my neighborhood	0	24.86	11.05	33.15	30.94	3	2.70
Item 6. There are indoor, air-conditioned places (shopping malls) where people can walk	0	20.99	11.60	27.07	40.33	3	2.87
*Aesthetics*							
Item 7. There are trees along the streets in my neighborhood	0	1.10	3.31	32.04	63.54	4	3.58
*Traffic safety*							
Item 8. There is so much traffic along nearby streets that it makes it difficult or unpleasant to walk in my neighborhood *	0	33.70	43.09	19.34	3.87	3 *	3.07 *
*Safety from crime*							
Item 9. There is a high crime rate in my neighborhood which makes it unsafe to go on walks during the day or at night *	0	55.80	32.04	7.18	4.97	4 *	3.39 *

* Reverse coded items/reverse scored.

**Table 3:** Walking time and physical activity according to tertiles of perceived neighborhood walkability * (*n* = 181).

Variable	All Participants	Perceived Neighborhood Walkability			*p*	*p_trend_*
		Lowest Tertile	Middle Tertile	Highest Tertile		
	(*n* = 181)	(*n* = 56)	(*n* = 68)	(*n* = 57)		
Walking time, hours in the past seven days						
<5.25	42 (23.20)	22 (39.29)	10 (14.71)	10 (17.54)	0.001	<0.001
5.25–10.49	77 (42.54)	24 (42.86)	33 (48.53)	20 (35.09)		
≥10.50	62 (34.25)	10 (17.86)	25 (36.76)	27 (47.37)		
Physical activity, hour/week						
<7.50	111 (61.33)	35 (62.50)	43 (63.24)	33 (57.89)	0.811	0.615
≥7.50	70 (38.67)	21 (37.50)	25 (36.76)	24 (42.11)		

* Tertiles cut-off values for the score of scale of perceived neighborhood walkability: 3.1111, 3.5556.

**Table 4:** Crude and adjusted associations of perceived neighborhood walkability * with measures of wellbeing and loneliness (*n* = 181).

Variable	All Participants	Perceived Neighborhood Walkability			β (*p*) ^†^	β (*p*) ^‡^
		Lowest Tertile	Middle Tertile	Highest Tertile		
	(*n* = 181)	(*n* = 56)	(*n* = 68)	(*n* = 57)		
Life satisfaction	7.68 ± 1.95	6.93 ± 2.42	7.82 ± 1.57	8.25 ± 1.60	0.658 (<0.001)	0.692 (0.002)
Happiness	7.94 ± 1.99	7.21 ± 2.53	8.10 ± 1.70	8.47 ± 1.44	0.629 (0.001)	0.718 (0.002)
Sense of purpose and meaning in life	8.07 ± 1.66	7.68 ± 1.93	8.13 ± 1.56	8.37 ± 1.41	0.345 (0.027)	0.214 (0.267)
Loneliness	1.64 ± 1.58	2.14 ± 1.79	1.63 ± 1.52	1.14 ± 1.27	−0.501 (0.001)	−0.458 (0.019)

* Tertiles cut-off values for the score of scale of perceived neighborhood walkability: 3.1111, 3.5556; ^†^ Crude models; ^‡^ Models adjusted for age, sex, marital status, education, employment, income, type of housing, length of residence, number of doctor-diagnosed chronic diseases, number of prescribed medications, smoking, alcohol intake, and number of negative life events.

**Table 5:** Crude and adjusted associations of individual items of perceived neighborhood walkability with measures of wellbeing and loneliness (*n* = 181).

Perceived Neighborhood Walkability	*n*	Life Satisfaction		Happiness		Sense of Purpose and Meaning in Life		Loneliness	
		β (*p*) *	β (*p*) ^†^	β (*p*) *	β (*p*) ^†^	β (*p*) *	β (*p*) ^†^	β (*p*) *	β (*p*) ^†^
**Land use mix-access**									
Item 1. There are many places to go within walking distance at my home									
Strongly disagree	23 (12.71) ^§^	−0.676 (0.118)	−0.823 (0.107)	−1.417 (0.002)	−1.767 (0.001)	−0.427 (0.256)	−0.446 (0.310)	0.748 (0.040)	0.443 (0.319)
Disagree	17 (9.39) ^§^	−1.582 (0.001)	−1.048 (0.058)	−1.005 (0.046)	−0.594 (0.280)	−1.179 (0.006)	−0.622 (0.190)	0.162 (0.693)	−0.149 (0.756)
Agree	33 (18.23)	−1.081 (0.004)	−1.064 (0.005)	−1.083 (0.005)	−1.081 (0.004)	−0.357 (0.273)	−0.190 (0.557)	0.544 (0.084)	0.498 (0.130)
Strongly agree	108 (59.67)	ref	ref	ref	ref	ref	ref	ref	ref
Item 2. The streets in my neighborhood are hilly, making my neighborhood difficult to walk in ^‡^									
Strongly disagree	87 (48.07)	ref	ref	ref	ref	ref	ref	ref	ref
Disagree	53 (29.28)	−0.854 (0.011)	−0.724 (0.037)	−0.157 (0.650)	−0.074 (0.835)	−0.319 (0.271)	−0.258 (0.378)	0.209 (0.447)	0.175 (0.558)
Agree	29 (16.02) ^§^	−0.805 (0.052)	−0.586 (0.158)	−0.862 (0.044)	−0.667 (0.118)	−0.115 (0.747)	0.025 (0.942)	0.690 (0.042)	0.342 (0.338)
Strongly agree	12 (6.63) ^§^	−0.330 (0.577)	0.016 (0.978)	−0.138 (0.821)	0.043 (0.944)	0.434 (0.396)	0.621 (0.219)	0.753 (0.122)	0.660 (0.200)
**Street connectivity**									
Item 3. There are many alternative routes for getting form place to place in my neighborhood									
Strongly disagree	10 (5.52) ^§^	−1.191 (0.057)	−0.313 (0.622)	−1.090 (0.094)	−0.177 (0.791)	−1.076 (0.048)	−0.326 (0.556)	0.526 (0.313)	−0.059 (0.917)
Disagree	6 (3.31) ^§^	−2.491 (0.002)	−2.892 (<0.001)	−1.356 (0.101)	−1.570 (0.054)	−1.109 (0.107)	−0.912 (0.176)	1.193 (0.072)	1.238 (0.071)
Agree	49 (27.07)	−0.604 (0.062)	−0.642 (0.040)	−0.516 (0.125)	−0.564 (0.085)	−0.419 (0.135)	−0.459 (0.091)	0.342 (0.204)	0.295 (0.284)
Strongly agree	116 (64.09)	ref	ref	ref	ref	ref	ref	ref	ref
**Infrastructure and safety for walking**									
Item 4. There are sidewalks on most of the streets in my neighborhood									
Strongly disagree	2 (1.10) ^§^	−2.931 (0.032)	−2.540 (0.062)	−2.762 (0.046)	−2.189 (0.111)	−0.785 (0.501)	−0.597 (0.603)	1.954 (0.084)	1.567 (0.179)
Disagree	4 (2.21) ^§^	−1.181 (0.224)	−0.820 (0.382)	−1.262 (0.199)	−0.736 (0.437)	−0.535 (0.519)	−0.296 (0.709)	0.954 (0.235)	0.889 (0.271)
Agree	45 (24.86)	−0.775 (0.020)	−0.434 (0.205)	−1.039 (0.002)	−0.845 (0.015)	−0.796 (0.005)	−0.456 (0.116)	0.187 (0.494)	−0.017 (0.954)
Strongly agree	130 (71.82)	ref	ref	ref	ref	ref	ref	ref	ref
Item 5. There are covered bridges in my neighborhood									
Strongly disagree	45 (24.86)	−0.488 (0.213)	−0.316 (0.564)	−0.899 (0.024)	−1.079 (0.052)	−0.455 (0.170)	−0.077 (0.867)	0.794 (0.011)	0.707 (0.127)
Disagree	20 (11.05) ^§^	−0.211 (0.679)	−0.292 (0.586)	0.029 (0.956)	−0.180 (0.738)	−0.361 (0.403)	−0.231 (0.606)	−0.439 (0.277)	−0.339 (0.451)
Agree	60 (33.15)	−0.261 (0.474)	−0.140 (0.703)	−0.471 (0.199)	−0.486 (0.188)	−0.577 (0.062)	−0.348 (0.257)	0.444 (0.124)	0.231 (0.454)
Strongly agree	56 (30.94)	ref	ref	ref	ref	ref	ref	ref	ref
Item 6. There are indoor, air-conditioned places (shopping malls) where people can walk									
Strongly disagree	38 (20.99)	−0.763 (0.051)	−0.904 (0.086)	−0.960 (0.016)	−1.462 (0.006)	−0.299 (0.369)	−0.118 (0.789)	0.842 (0.008)	0.730 (0.105)
Disagree	21 (11.60) ^§^	−0.571 (0.235)	−0.051 (0.921)	−0.472 (0.334)	−0.264 (0.607)	−0.199 (0.629)	0.463 (0.284)	0.562 (0.148)	0.486 (0.266)
Agree	49 (27.07)	−0.347 (0.334)	−0.279 (0.454)	−0.472 (0.196)	−0.505 (0.178)	−0.349 (0.258)	−0.078 (0.803)	0.188 (0.515)	0.044 (0.890)
Strongly agree	73 (40.33)	ref	ref	ref	ref	ref	ref	ref	ref
**Aesthetics**									
Item 7. There are trees along the streets in my neighborhood									
Strongly disagree	2 (1.10) ^§^	−5.422 (<0.001)	−2.260 (0.231)	−3.200 (0.021)	1.802 (0.348)	−3.743 (<0.001)	0.253 (0.874)	1.048 (0.353)	−0.392 (0.811)
Disagree	6 (3.31) ^§^	1.078 (0.164)	1.236 (0.158)	1.133 (0.161)	0.824 (0.354)	0.923 (0.169)	0.583 (0.432)	0.548 (0.408)	0.472 (0.534)
Agree	58 (32.04)	−0.680 (0.023)	−0.509 (0.096)	−0.803 (0.010)	−0.697 (0.025)	−0.519 (0.045)	−0.403 (0.120)	0.479 (0.061)	0.307 (0.246)
Strongly agree	115 (63.54)	ref	ref	ref	ref	ref	ref	ref	ref
**Traffic safety**									
Item 8. There is so much traffic along nearby streets that it makes it difficult or unpleasant to walk in my neighborhood ^‡^									
Strongly disagree	61 (33.70)	ref	ref	ref	ref	ref	ref	ref	ref
Disagree	78 (43.09)	−1.016 (0.002)	−0.893 (0.007)	−1.086 (0.001)	−0.960 (0.004)	−1.137 (<0.001)	−1.088 (<0.001)	0.315 (0.246)	0.131 (0.650)
Agree	35 (19.34)	−1.426 (<0.001)	−1.412 (0.001)	−1.031 (0.012)	−1.023 (0.016)	−1.175 (0.001)	−1.384 (<0.001)	0.480 (0.155)	0.060 (0.870)
Strongly agree	7 (3.87) ^§^	−0.855 (0.256)	−0.402 (0.595)	−1.974 (0.011)	−1.565 (0.045)	−0.518 (0.412)	−0.279 (0.651)	0.766 (0.227)	0.491 (0.465)
**Safety from crime**									
Item 9. There is a high crime rate in my neighborhood which makes it unsafe to go on walks during the day or at night ^‡^									
Strongly disagree	101 (55.80)	ref	ref	Ref	ref	ref	ref	ref	ref
Disagree	58 (32.04)	−0.818 (0.010)	−0.555 (0.072)	−0.824 (0.012)	−0.634 (0.046)	−0.640 (0.019)	−0.397 (0.129)	0.141 (0.583)	−0.068 (0.794)
Agree	13 (7.18) ^§^	−0.829 (0.143)	−0.818 (0.150)	−0.084 (0.885)	0.198 (0.734)	−0.808 (0.096)	−0.837 (0.083)	1.247 (0.007)	1.218 (0.011)
Strongly agree	9 (4.97) ^§^	−1.171 (0.080)	−1.558 (0.027)	−0.460 (0.502)	−0.803 (0.266)	−0.347 (0.544)	−0.338 (0.570)	1.110 (0.041)	1.437 (0.016)

* Crude models; ^†^ Model adjusted for age, sex, marital status, education, employment, income, type of housing, length of residence, number of doctor-diagnosed chronic diseases, number of prescribed medications, smoking, alcohol intake, and number of negative life events; ^‡^ Reverse coded items/reverse scored; ^§^ Estimates based on few than 30 observations are considered unreliable.

## Appendix A

**Table A1:** Perceived neighborhood walkability: The reduced version vs. Chinese NEWS-A (*n* = 46).

Sub-Scales	The Reduced Version		Chinese NEWS-A Sub-Scales			
	No. of Item	Mean ± SD	No. of Item	Mean ± SD	*r* ^†^	*p* ^‡^
Land use mix-access	2	3.17 ± 0.85	6	3.27 ± 0.54	0.723 ***	0.463
Street connectivity	1	3.59 ± 0.83	3	3.46 ± 0.59	0.456 **	0.284
Infrastructure and safety for walking	3	3.07 ± 0.79	6	3.12 ± 0.35	0.420 **	0.609
Aesthetics	1	3.57 ± 0.62	4	2.62 ± 0.64	0.444 **	<0.001
Traffic safety	1	3.02 ± 0.86	3	2.96 ± 0.62	0.646 ***	0.552
Safety from crime	1	3.41 ± 0.72	3	3.51 ± 0.61	0.522 ***	0.299
Mean walkability score	9	3.24 ± 0.54	36	3.16 ± 0.35	0.764 ***	0.095
Cronbach’s α		0.776		0.652		

** *p*-value < 0.01, *** *p*-value < 0.001. ^†^ Pearson correlation coefficient (*r*) and the corresponding *p*-value were obtained using Pearson correlations. ^‡^ *p*-values were obtained using paired-*t* tests.
